# *catena*-Poly[[aqua­copper(II)]-μ_2_-imino­diacetato-κ^4^ *O*,*N*,*O*′:*O*′]

**DOI:** 10.1107/S1600536811041286

**Published:** 2011-10-12

**Authors:** Qin Zhong, Yu-Hong Wang, Xue-Ting Zhang

**Affiliations:** a School of Chemistry and Bioengineering, Suzhou University of Science and Technology, Suzhou 215009, People’s Republic of China

## Abstract

In the title compound, [Cu(C_4_H_5_O_4_)(H_2_O)]_*n*_, the imino­diacetate (ida) ligands link the Cu^II^ atoms into polymeric zigzag chains running along [010]. Each Cu^II^ ion is five-coordinated in a distorted square-pyramidal geometry by one N and two O atoms from an ida ligand, one O atom from the neighbouring ida ligand and one water O atom. In the crystal, the polymeric chains are held together *via* inter­molecular O—H⋯O and N—H⋯O hydrogen bonds.

## Related literature

For applications of coordination polymers containing bridging carboxyl­ate groups, see: Dey *et al.* (2003 ▶); Wu *et al.* (2009 ▶); Zhang *et al.* (2008 ▶). For coordination polymers with imino­diacetic acid, see: Bresciani-Pahor *et al.* (1984 ▶); Ren *et al.* (2003 ▶); Song *et al.* (2011 ▶).
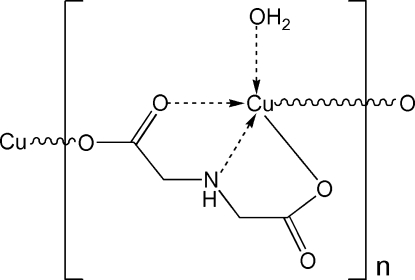


## Experimental

### 

#### Crystal data

[Cu(C_4_H_5_O_4_)(H_2_O)]
*M* *_r_* = 212.65
Monoclinic, 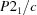

*a* = 6.563 (3) Å
*b* = 9.870 (4) Å
*c* = 10.876 (4) Å
β = 99.802 (8)°
*V* = 694.2 (5) Å^3^
*Z* = 4
Mo *K*α radiation
μ = 3.12 mm^−1^
*T* = 223 K
0.40 × 0.25 × 0.15 mm

#### Data collection

Rigaku Saturn diffractometer
Absorption correction: multi-scan (*REQAB*; Jacobson, 1998 ▶) *T*_min_ = 0.369, *T*_max_ = 0.652
3854 measured reflections
1571 independent reflections
1358 reflections with *I* > 2σ(*I*)
*R*_int_ = 0.026

#### Refinement

*R*[*F* ^2^ > 2σ(*F* ^2^)] = 0.034
*wR*(*F* ^2^) = 0.082
*S* = 1.02
1571 reflections
110 parameters
3 restraints
H atoms treated by a mixture of independent and constrained refinement
Δρ_max_ = 0.45 e Å^−3^
Δρ_min_ = −0.48 e Å^−3^

### 

Data collection: *CrystalClear* (Rigaku, 2001 ▶); cell refinement: *CrystalClear*; data reduction: *CrystalStructure* (Rigaku, 2001 ▶); program(s) used to solve structure: *SHELXS97* (Sheldrick, 2008 ▶); program(s) used to refine structure: *SHELXL97* (Sheldrick, 2008 ▶); molecular graphics: *SHELXTL* (Sheldrick, 2008 ▶); software used to prepare material for publication: *SHELXTL*.

## Supplementary Material

Crystal structure: contains datablock(s) I, global. DOI: 10.1107/S1600536811041286/cv5163sup1.cif

Structure factors: contains datablock(s) I. DOI: 10.1107/S1600536811041286/cv5163Isup2.hkl

Additional supplementary materials:  crystallographic information; 3D view; checkCIF report

## Figures and Tables

**Table 1:** Hydrogen-bond geometry (Å, °)

*D*—H⋯*A*	*D*—H	H⋯*A*	*D*⋯*A*	*D*—H⋯*A*
O5—H5*A*⋯O1^i^	0.87 (1)	2.08 (1)	2.936 (4)	168 (4)
O5—H5*B*⋯O2^ii^	0.87 (1)	1.99 (1)	2.860 (4)	171 (4)
N1—H11*A*⋯O2^i^	0.86 (1)	2.13 (1)	2.992 (3)	173 (3)

Symmetry codes: (i) 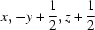; (ii) 
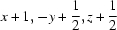
.

**Fig. 1.**
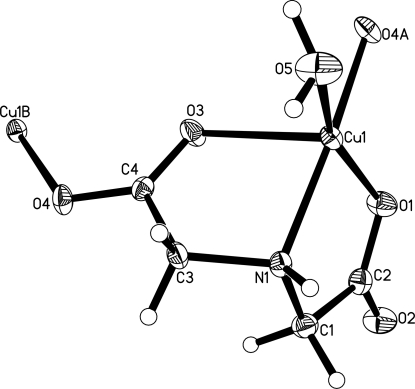
A portion of the crystal structure of (I), showing the atomic numbering and 30% probabilty displacement ellipsoids [symmetry codes: (A) -x + 2, y - 1/2, -z + 3/2; (B) -x + 2, y + 1/2, -z + 3/2]

**Fig. 2.**
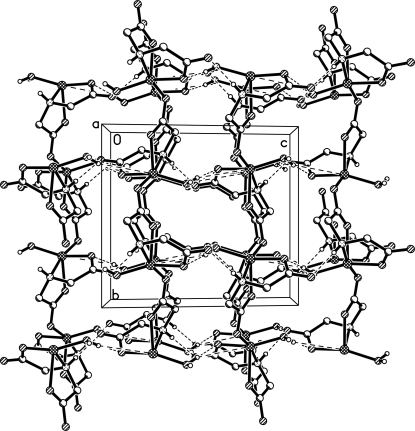
A portion of the crystal packing viewed approximately down the a axis. Dashed lines denote hydrogen bonds. H atoms with no hydrogen bond interactions have been omitted for clarity.

## supplementary crystallographic information

### Comment

The syntheses of coordination polymers containing bridging carboxylate groups are of current interest due to potential applications in the areas of magnetism, ion exchange and photochemistry (Dey *et al.*, 2003; Wu *et al.*, 2009; Zhang *et al.*, 2008). The iminodiacetic acid has been found to be useful ligand, and a lot of transition metal polymers of iminodiacetic acid have been reported (Bresciani-Pahor *et al.*, 1984; Ren *et al.*, 2003; Song *et al.*, 2011). Here, we report the crystal structure of the title compound, (I), a one-dimensional Cu(II) coordination polymer obtained by the hydrothermal synthesis reaction of iminodiacetic acid and copper(II) chlorine.

The title complex (I) is a one-dimensional zigzag chain coordination polymer, which results from the fact that the copper(II) ions are bridged sequentially by *syn-anti* carboxylate groups. A perspective view of the mononuclear fragment of (I) is given in Fig. 1. Each copper(II) ion is in a distorted square pyramidal geometry with three donor atoms (O1, N1, O3) of the ida ligand, one oxygen atoms O4A (A -*x* + 2, *y* - 1/2, -*z* + 3/2) belonging to the carboxylate group of one adjacent ida ligand and one terminal O (O5) atom of H_2_O molecule. Two five-membered chelate rings [–Cu1—O3—C4—C3—N1- and –Cu1—O1—C2—C1—N1-] are formed with the metal atoms, and the two fused ring systems are folded along the common Cu1—N1 axis by 101.5 (1)°. In (I), each ida ligand is tetradentate when the bridge involving atom O4A is considered. One of carboxylate groups of each ida ligand is in an *syn-anti* conformation with respect to the two copper centres. Thus, the carboxylate groups act as bridges and connect the copper(II) centers to form a 1-D zigzag chain coordination polymer.

The one-dimensional polymeric chains are packed through intermolecular O—H···O and N—H···O hydrogen bonds (Table 1) to form three-dimensional structure (Fig. 2).

### Experimental

CuCl_2_^.^2H_2_O (0.0171 g, 0.1 mmol), iminodiacetic acid (0.0133 g, 0.1 mmol), NaOH (0.0084 g, 0.2 mmol), H_2_O (0.5 mL) and ethanol (3 mL) were placed in a thick Pyrex tube and heated at 120°C for 3 days. After cooling at a rate of 5°C/h to the ambient temperature, blue block crystals were collected, washed with anhydrous ethanol, and then dried at room temperature. The yield is 76% based on iminodiacetic acid. Analysis found: C, 22.98; H, 3.36; N, 6.56%. Calculated for C_4_H_7_CuNO_5_: C, 22.59; H, 3.32; N, 6.59%.

### Refinement

C-bound H atoms were geometrically positioned and refinded using a riding model, with *U*_iso_(H) = 1.2 *U*_eq_(C) [*d*(C—H) = 0.98Å (for CH_2_)]. H atoms attached to N and O were located on difference maps and refined with N—H distances restrained to 0.87 (1)Å (*U*_iso_(H) = 1.2 *U*_eq_(N)), and with O—H distances retsrained to 0.86 (1) Å (*U*_iso_(H) = 1.2 *U*_eq_(O)).

### Crystal data

**Table:**

[Cu(C_4_H_5_O_4_)(H_2_O)]	*F*(000) = 428
*M**_r_* = 212.65	*D*_x_ = 2.035 Mg m^−^^3^
Monoclinic, *P*2_1_/*c*	Mo *K*α radiation, λ = 0.71075 Å
Hall symbol: -P 2ybc	Cell parameters from 3372 reflections
*a* = 6.563 (3) Å	θ = 3.1–27.5°
*b* = 9.870 (4) Å	µ = 3.12 mm^−^^1^
*c* = 10.876 (4) Å	*T* = 223 K
β = 99.802 (8)°	Block, blue
*V* = 694.2 (5) Å^3^	0.40 × 0.25 × 0.15 mm
*Z* = 4	

### Data collection

**Table:**

Rigaku Saturn diffractometer	1571 independent reflections
Radiation source: fine-focus sealed tube	1358 reflections with *I* > 2σ(*I*)
graphite	*R*_int_ = 0.026
Detector resolution: 14.63 pixels mm^-1^	θ_max_ = 27.5°, θ_min_ = 3.2°
ω scans	*h* = −8→8
Absorption correction: multi-scan (*REQAB*; Jacobson, 1998)	*k* = −12→12
*T*_min_ = 0.369, *T*_max_ = 0.652	*l* = −9→14
3854 measured reflections	

### Refinement

**Table:**

Refinement on *F*^2^	Primary atom site location: structure-invariant direct methods
Least-squares matrix: full	Secondary atom site location: difference Fourier map
*R*[*F*^2^ > 2σ(*F*^2^)] = 0.034	Hydrogen site location: inferred from neighbouring sites
*wR*(*F*^2^) = 0.082	H atoms treated by a mixture of independent and constrained refinement
*S* = 1.02	*w* = 1/[σ^2^(*F*_o_^2^) + (0.046*P*)^2^ + 0.157*P*] where *P* = (*F*_o_^2^ + 2*F*_c_^2^)/3
1571 reflections	(Δ/σ)_max_ < 0.001
110 parameters	Δρ_max_ = 0.45 e Å^−^^3^
3 restraints	Δρ_min_ = −0.48 e Å^−^^3^

### Special details

**Table:**

Geometry. All e.s.d.'s (except the e.s.d. in the dihedral angle between two l.s. planes) are estimated using the full covariance matrix. The cell e.s.d.'s are taken into account individually in the estimation of e.s.d.'s in distances, angles and torsion angles; correlations between e.s.d.'s in cell parameters are only used when they are defined by crystal symmetry. An approximate (isotropic) treatment of cell e.s.d.'s is used for estimating e.s.d.'s involving l.s. planes.
Refinement. Refinement of *F*^2^ against ALL reflections. The weighted *R*-factor *wR* and goodness of fit *S* are based on *F*^2^, conventional *R*-factors *R* are based on *F*, with *F* set to zero for negative *F*^2^. The threshold expression of *F*^2^ > σ(*F*^2^) is used only for calculating *R*-factors(gt) *etc*. and is not relevant to the choice of reflections for refinement. *R*-factors based on *F*^2^ are statistically about twice as large as those based on *F*, and *R*- factors based on ALL data will be even larger.

### Fractional atomic coordinates and isotropic or equivalent isotropic displacement parameters (Å^2^)

**Table:**

	*x*	*y*	*z*	*U*_iso_*/*U*_eq_	
Cu1	0.85030 (5)	0.23532 (3)	0.75251 (3)	0.02015 (14)	
O1	0.6802 (4)	0.2321 (2)	0.5873 (2)	0.0312 (5)	
O2	0.4101 (3)	0.3228 (3)	0.4674 (2)	0.0378 (6)	
O3	0.9803 (3)	0.4446 (2)	0.7449 (2)	0.0312 (5)	
O4	0.9274 (3)	0.64968 (19)	0.8186 (2)	0.0265 (5)	
O5	0.9679 (4)	0.1781 (3)	0.9246 (2)	0.0454 (6)	
H5A	0.897 (6)	0.212 (4)	0.978 (3)	0.054*	
H5B	1.1022 (18)	0.184 (5)	0.945 (4)	0.054*	
N1	0.6164 (4)	0.3497 (2)	0.7986 (2)	0.0200 (5)	
H11A	0.546 (4)	0.303 (3)	0.844 (3)	0.024*	
C1	0.4669 (4)	0.3788 (3)	0.6828 (3)	0.0263 (6)	
H1A	0.4639	0.4767	0.6674	0.032*	
H1B	0.3282	0.3511	0.6949	0.032*	
C2	0.5202 (5)	0.3069 (3)	0.5707 (3)	0.0256 (6)	
C3	0.7073 (4)	0.4735 (3)	0.8622 (3)	0.0226 (6)	
H3A	0.7568	0.4537	0.9505	0.027*	
H3B	0.6011	0.5440	0.8571	0.027*	
C4	0.8864 (4)	0.5246 (3)	0.8023 (3)	0.0208 (6)	

### Atomic displacement parameters (Å^2^)

**Table:**

	*U*^11^	*U*^22^	*U*^33^	*U*^12^	*U*^13^	*U*^23^
Cu1	0.0235 (2)	0.0177 (2)	0.0204 (2)	0.00209 (13)	0.00672 (14)	0.00000 (13)
O1	0.0320 (12)	0.0387 (12)	0.0226 (12)	0.0087 (10)	0.0042 (9)	−0.0061 (9)
O2	0.0342 (12)	0.0540 (14)	0.0233 (13)	0.0085 (11)	−0.0003 (10)	−0.0008 (11)
O3	0.0306 (12)	0.0195 (10)	0.0491 (15)	−0.0004 (9)	0.0229 (10)	−0.0010 (9)
O4	0.0341 (11)	0.0187 (9)	0.0297 (12)	−0.0081 (9)	0.0138 (9)	−0.0055 (8)
O5	0.0394 (14)	0.0669 (18)	0.0307 (15)	0.0129 (14)	0.0085 (11)	0.0028 (12)
N1	0.0231 (12)	0.0185 (11)	0.0202 (13)	−0.0033 (10)	0.0085 (9)	−0.0004 (9)
C1	0.0233 (14)	0.0289 (15)	0.0263 (17)	0.0006 (13)	0.0030 (12)	−0.0012 (12)
C2	0.0284 (16)	0.0253 (14)	0.0235 (16)	−0.0030 (13)	0.0055 (12)	0.0000 (12)
C3	0.0265 (15)	0.0171 (12)	0.0263 (16)	−0.0019 (11)	0.0101 (12)	−0.0053 (11)
C4	0.0203 (14)	0.0199 (13)	0.0215 (15)	−0.0032 (11)	0.0016 (11)	0.0019 (11)

### Geometric parameters (Å, °)

**Table:**

Cu1—O1	1.948 (2)	O5—H5B	0.873 (10)
Cu1—O4^i^	1.955 (2)	N1—C3	1.478 (3)
Cu1—O5	1.981 (3)	N1—C1	1.486 (4)
Cu1—N1	2.036 (2)	N1—H11A	0.863 (10)
Cu1—O3	2.241 (2)	C1—C2	1.503 (4)
O1—C2	1.271 (4)	C1—H1A	0.9800
O2—C2	1.238 (4)	C1—H1B	0.9800
O3—C4	1.234 (3)	C3—C4	1.523 (4)
O4—C4	1.270 (3)	C3—H3A	0.9800
O4—Cu1^ii^	1.955 (2)	C3—H3B	0.9800
O5—H5A	0.873 (10)		
			
O1—Cu1—O4^i^	88.70 (10)	C1—N1—H11A	104 (2)
O1—Cu1—O5	159.50 (11)	Cu1—N1—H11A	110 (2)
O4^i^—Cu1—O5	93.05 (10)	N1—C1—C2	112.6 (2)
O1—Cu1—N1	84.15 (10)	N1—C1—H1A	109.1
O4^i^—Cu1—N1	168.97 (9)	C2—C1—H1A	109.1
O5—Cu1—N1	96.58 (10)	N1—C1—H1B	109.1
O1—Cu1—O3	98.23 (9)	C2—C1—H1B	109.1
O4^i^—Cu1—O3	93.97 (8)	H1A—C1—H1B	107.8
O5—Cu1—O3	102.01 (11)	O2—C2—O1	122.9 (3)
N1—Cu1—O3	78.80 (8)	O2—C2—C1	119.7 (3)
C2—O1—Cu1	116.8 (2)	O1—C2—C1	117.4 (3)
C4—O3—Cu1	110.15 (17)	N1—C3—C4	110.7 (2)
C4—O4—Cu1^ii^	121.34 (19)	N1—C3—H3A	109.5
Cu1—O5—H5A	111 (3)	C4—C3—H3A	109.5
Cu1—O5—H5B	116 (3)	N1—C3—H3B	109.5
H5A—O5—H5B	116 (4)	C4—C3—H3B	109.5
C3—N1—C1	113.1 (2)	H3A—C3—H3B	108.1
C3—N1—Cu1	108.17 (17)	O3—C4—O4	125.4 (3)
C1—N1—Cu1	108.40 (17)	O3—C4—C3	119.5 (2)
C3—N1—H11A	113 (2)	O4—C4—C3	115.0 (2)
			
O4^i^—Cu1—O1—C2	−164.3 (2)	O3—Cu1—N1—C1	93.24 (18)
O5—Cu1—O1—C2	100.5 (3)	C3—N1—C1—C2	125.0 (3)
N1—Cu1—O1—C2	7.3 (2)	Cu1—N1—C1—C2	5.1 (3)
O3—Cu1—O1—C2	−70.5 (2)	Cu1—O1—C2—O2	173.5 (2)
O1—Cu1—O3—C4	100.6 (2)	Cu1—O1—C2—C1	−6.1 (3)
O4^i^—Cu1—O3—C4	−170.2 (2)	N1—C1—C2—O2	−179.3 (3)
O5—Cu1—O3—C4	−76.2 (2)	N1—C1—C2—O1	0.3 (4)
N1—Cu1—O3—C4	18.2 (2)	C1—N1—C3—C4	−82.1 (3)
O1—Cu1—N1—C3	−129.37 (18)	Cu1—N1—C3—C4	38.0 (3)
O4^i^—Cu1—N1—C3	−79.5 (5)	Cu1—O3—C4—O4	177.6 (2)
O5—Cu1—N1—C3	71.24 (19)	Cu1—O3—C4—C3	−1.3 (3)
O3—Cu1—N1—C3	−29.75 (17)	Cu1^ii^—O4—C4—O3	1.8 (4)
O1—Cu1—N1—C1	−6.38 (17)	Cu1^ii^—O4—C4—C3	−179.27 (19)
O4^i^—Cu1—N1—C1	43.5 (5)	N1—C3—C4—O3	−24.3 (4)
O5—Cu1—N1—C1	−165.77 (18)	N1—C3—C4—O4	156.6 (2)

Symmetry codes: (i) −*x*+2, *y*−1/2, −*z*+3/2; (ii) −*x*+2, *y*+1/2, −*z*+3/2.

### Hydrogen-bond geometry (Å, °)

**Table:**

*D*—H···*A*	*D*—H	H···*A*	*D*···*A*	*D*—H···*A*
O5—H5A···O1^iii^	0.87 (1)	2.08 (1)	2.936 (4)	168 (4)
O5—H5B···O2^iv^	0.87 (1)	1.99 (1)	2.860 (4)	171 (4)
N1—H11A···O2^iii^	0.86 (1)	2.13 (1)	2.992 (3)	173 (3)

Symmetry codes: (iii) *x*, −*y*+1/2, *z*+1/2; (iv) *x*+1, −*y*+1/2, *z*+1/2.
